# Renal Nerve Activity and Arterial Depressor Responses Induced by Neuromodulation of the Deep Peroneal Nerve in Spontaneously Hypertensive Rats

**DOI:** 10.3389/fnins.2022.726467

**Published:** 2022-05-16

**Authors:** Maria Alejandra Gonzalez-Gonzalez, Kevin Romero, John Beitter, David Lloyd, Danny V. Lam, Ana Guadalupe Hernandez-Reynoso, Aswini Kanneganti, Han-Kyul Kim, Caroline K. Bjune, Scott Smith, Wanpen Vongpatanasin, Mario I. Romero-Ortega

**Affiliations:** ^1^Department of Biomedical Engineering and Biomedical Sciences, University of Houston, Houston, TX, United States; ^2^Department of Biomedical Engineering, The University of Texas at Dallas, Dallas, TX, United States; ^3^Hypertension Section, Department of Internal Medicine, University of Texas Southwestern Medical Center, Dallas, TX, United States; ^4^Draper Laboratory, Boston, MA, United States; ^5^Applied Clinical Research, University of Texas Southwestern Medical Center, Dallas, TX, United States

**Keywords:** hypertension, neuromodulation, spontaneously hypertensive rats, renal nerve activity, deep peroneal nerve

## Abstract

Hypertension is a main cause of death in the United States with more than 103 million adults affected. While pharmacological treatments are effective, blood pressure (BP) remains uncontrolled in 50–60% of resistant hypertensive subjects. Using a custom-wired miniature electrode, we previously reported that deep peroneal nerve stimulation (DPNS) elicited acute cardiovascular depressor responses in anesthetized spontaneously hypertensive rats (SHRs). Here, we further study this effect by implementing a wireless system and exploring different stimulation parameters to achieve a maximum depressor response. Our results indicate that DPNS consistently induces a reduction in BP and suggests that renal sympathetic nerve activity (RSNA) is altered by this bioelectronic treatment. To test the acute effect of DPNS in awake animals, we developed a novel miniaturized wireless microchannel electrode (w-μCE), with a Z-shaped microchannel through which the target nerves slide and lock into the recording/stimulation chamber. Animals implanted with w-μCE and BP telemetry systems for 3 weeks showed an average BP of 150 ± 14 mmHg, which was reduced significantly by an active DPNS session to 135 ± 8 mmHg (*p* < 0.04), but not in sham-treated animals. The depressor response in animals with an active w-μCE was progressively returned to baseline levels 14 min later (164 ± 26 mmHg). This depressor response was confirmed in restrained fully awake animals that received DPNS for 10 days, where tail-cuff BP measurements showed that systolic BP in SHR lowered 10% at 1 h and 16% 2 h after the DPNS when compared to the post-implantation baseline. Together, these results support the use of DPN neuromodulation as a possible strategy to lower BP in drug-resistant hypertension.

## Introduction

Hypertension is an important risk factor in the development of cardiovascular and kidney disease and stroke and heart failure, affecting more than 103.3 million people in the United States (Muntner et al., 2020). The American College of Cardiology/American Heart Association 2018 guidelines classified average systolic blood pressure (BP) > 130 mmHg and diastolic BP > 80 mmHg, on at least two separate occasions, as hypertension (Flack et al., 2018). Broadly used pharmaceuticals for this condition include renin-angiotensin inhibitors, angiotensin-converting enzyme inhibitors, and angiotensin II receptor blockers. Others include thiazide diuretics, beta-blockers, and calcium antagonists, depending on co-morbidities (Mendoza et al., 2021). Unfortunately, despite the use of multiple antihypertensive drugs in combination, BP remains poorly controlled in 50–60% of the hypertensive population (Carey et al., 2018), and approximately 12–18% of them develop resistant hypertension (RH); defined as BP > 140/90 mmHg despite the use of three antihypertensive drugs of complementary mechanisms, including a diuretic agent (Chia et al., 2020). Alternative treatments aim at reducing the renal sympathetic tone, or the hyper-reflex sympathetic signals from the carotid body, which are sensitive to oxygen and blood flow. For the former, renal denervation has been shown to reduce mean arterial pressure (MAP) in six placebo-controlled trials (systolic BP−5.53 mmHg), although with high variability, and the possible kidney reinnervation (Lauder et al., 2020). For the latter, unilateral resection of the carotid body in 15 patients with RH was shown to reduce ambulatory BP in 8 subjects, although serious adverse events were reported in two of them (Narkiewicz et al., 2016).

Several medical devices have been developed for RH (Lauder et al., 2020), such as the Rheos (CVRx) system, with bilateral electrodes implanted near the aortic arch to stimulate the baroreceptors, which exert an inhibitory influence on sympathetic nerve activity (Groenland and Spiering, 2020). This baroreflex activation therapy was reported in an open-label study to reduce in-office MAP in patients with RH, but not ambulatory RH (Bakris et al., 2012). In addition, a double-blind, randomized pivotal study with 265 patients was failed to meet efficacy endpoints, and 25% showed adverse surgical complications (Ewen et al., 2017). A second generation of the Rheos device has provided promising results, although side effects due to high stimulation intensities remain (Mahfoud et al., 2021).

Alternative treatments include electroacupuncture based either on Qi hypotheses or mechanistic information, where controlled clinical trials have shown a significantly lower 24-h ambulatory MAP (>6 mmHg), correlated with reductions in circulating norepinephrine, renin, and aldosterone (Flachskampf et al., 2007; Li et al., 2015). We previously reported that stimulation of the deep peroneal nerve (DPNS), a fascicle of the sciatic nerve in proximity to the acupuncture point ST-36, induced a 23 mmHg reduction in MAP in anesthetized spontaneously hypertensive rats (SHRs) (Kim and Romero-Ortega, 2012; Kim et al., 2014). However, confirming the depressor effect in freely moving animals who underwent DPNS has remained a challenge, given the small size of the rat peroneal nerve (≈200 μm OD) and the need for fully implantable miniature wireless stimulators to avoid nerve injury and/or discomfort to the animal during ambulation. Here, we report studies that refined stimulation parameters for optimal MAP reduction by DPNS in SHR animals. We previously developed a miniature wireless microchannel electrode (w-μCE) with inductive stimulator circuit and an external power/command control (Freeman et al., 2017; Hernandez-Reynoso et al., 2019), here we improved it with a novel nerve attachment microchannel that facilitates implantation and anchor of the device into small nerves and allowed sub-chronic DPNS in awake animals. The results support the notion that wireless neuromodulation devices can be used to deliver bioelectronic treatment to lower MAP in a rat model of hypertension and show promise as a treatment for RH.

## Materials and Methods

### Animals

**FIGURE 1:**
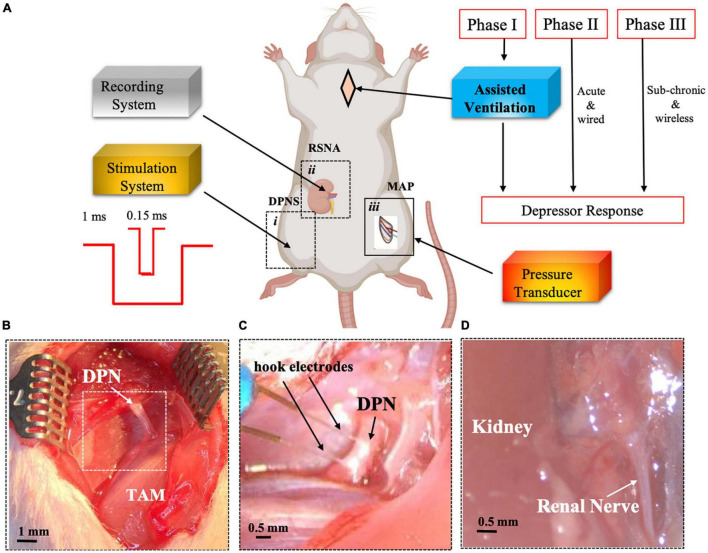
Experimental set-up for DPNS to modulate MAP. Adult SRH rats were anesthetized. Phase I animals received assisted ventilation (*n* = 5), and phase II did not (*n* = 8), and phase III the MAP was measured by telemetry and DPNS with a wireless system (*n* = 11). The effect of DPNS on RSNA and MAP was evaluated, **(A)** Animal setup: a stimulator system with a pulse generator for short (0.15 ms) or long (1 ms) pulses was connected to a hook electrode placed on the DPN *(i).* A second electrode was placed on the renal nerve to record neural activity *(ii).* A catheter was implanted in the femoral artery and connected to a pressure transducer to monitor MAP *(iii).* Dotted-line inserts *(i* and *ii)* show the site where pictures in panels **(B–D)** were obtained. Photographs of the DPN before **(B)** and after **(C)** electrode implantation are displayed. Insert in panel **B** is magnified in panel **C** after electrode placement. **(D)** Identification of renal nerve. SHR, spontaneous hypertensive rats; RSNA, renal sympathetic nerve activity; DPNS, Deep peroneal nerve stimulation; DPN, deep peroneal nerve: MAP, mean arterial pressure; TAM, tibialis anterior muscle.

A total of 26 adult male SHRs (300–350 g, 12–14 weeks old, Charles River, Wilmington, MA, United States) were used in this study. SHRs are considered an established model of primary hypertension due to an overactivated sympathetic drive (Shanks et al., 2013; Gu et al., 2020). The animals were divided into three separate study phase cohorts, which are as follows: (I) evaluation of acute pulse duration effect in anesthetized breathing-assisted rats (*n* = 5), (II) confirmation of optimized stimulation parameters in anesthetized spontaneous breathing animals (*n* = 8; Figure 1), and (III) testing sub-chronic DPNS in awake animals (*n* = 13). Animals were anesthetized with vaporized isoflurane (2%) in a constant oxygen flux (2 L/min) delivered by a calibrated vaporizer, placed on a warm pad, and body temperature, cardiac rate, and respiration were monitored constantly. Nerve stimulation in Phase I and II cohorts was done using a wired hook platinum electrode, whereas Phase III evaluation used fully implantable w-μCE stimulators and included a sham group implanted with non-functional electrodes.

### Tracheotomy

Animals participating in Phase I of the study underwent a tracheotomy for assisted mechanical ventilation. The trachea was exposed by a midline incision in the neck (1.5–2 cm), and an endotracheal tube (1.72 mm diameter) was inserted into the airway. A respirator (Model 683, Harvard Apparatus) was used with a tidal volume of 2.5 ml/breath and respiratory rate of 70 breaths/min throughout the experiment.

### Deep Peroneal Nerve Stimulation

A 2.0 cm incision on the hind-limb below the femur exposed the biceps femoris and vastus lateralis muscles, which were bluntly dissected to expose the DPN branch from the sciatic nerve and gently isolated from surrounding connective tissue using a glass rod and kept hydrated with warm physiological saline solution (pH 7.2). Either wired hook platinum electrodes (Phase I and II studies) or a wireless stimulator (Phase III studies) were used. For Phase III studies, the wound was closed after implantation of the w-μCE electrode onto the DPN, topical antibiotics were applied, and antibiotic and analgesic were administrated (cefazolin, 5 mg/kg and buprenorphine sustained-release [SR], 1 mg/kg; respectively). These animals were recovered for 3 weeks before applying wireless DPN neuromodulation.

#### Stimulation Parameters in Acute Studies

Stimulation parameters were evaluated for maximum depressor responses, using either voltage (0.2–1.2 mV, *n* = 11 tests; 2 animals) or current (0.06–2.0 mA, *n* = 17 tests; 3 animals) 0.15 and 1.0 ms cathodic monophasic pulses, applied with a PlexStim Electrical Stimulator System (Plexon Inc). The effect was confirmed by the evoked contraction of the tibialis anterior (TA) muscle and digits, which was videotaped and used to determine the threshold and effectiveness of DPN stimulation. The frequency for pulses was maintained at 2 Hz for 30 ± 15 s. The effect of DPN stimulation on the blood pressure was evaluated by implanting an arterial pressure sensor.

#### Sub-Chronic Deep Peroneal Nerve Neuromodulation

A miniature implantable w-μCE neural stimulator was developed using a sub-millimeter radiofrequency (RF) stimulator circuit (150-turn coil with a Nickel-Zinc ferrite core, a 7.0 pF capacitor tuned at 10.9 MHz, a Schottky diode, and a 100 pF shunt capacitor (Hernandez-Reynoso et al., 2019)), integrated into a 3D printed nerve-attachment device with a Z-shaped microchannel, leading to a stimulation chamber containing platinum trace electrodes. This new design facilitates nerve implantation by placing it underneath the nerve and gently lifting it over the microchannel. This causes a transient longitudinal elongation and transverse compression of the nerve, which then allows it to pass through a smaller microchannel (internal diameter 20–30% less than the DPN diameter) and into the electrode chamber. Lowering the device, release the tension in the nerve allowing it to expand to its original size and locking it in place. Seven electrodes were used for this study and were electrochemically characterized before use, with an average impedance value of 250.63 ± 53.42 KΩ at 1 kHz frequency. During stimulation, we used an external RF antenna, with an electrical field ranging from 16.87 to 27.5 A/m, at 2–10 cm from the implanted w-μCE stimulator. The limits of exposure to electromagnetic fields were established to meet the Federal Communications Commission (Bassen et al., 2005). Three weeks after recovery, animals implanted with w-μCE stimulators onto the DPN were stimulated for 8 min with 200 mV square monophasic pulses (200 μs) at 2 Hz, using a pulse generator (Agilent 81110A) that was connected to an external RF amplifier, and a transmission antenna with a 10.0 MHz carrier frequency (AG 1012, T&C Power Conversion, Inc).

### Renal Sympathetic Nerve Activity (RSNA)

A 2.5 cm midline abdominal incision was made to visualize the kidney. Using a dissecting microscope, a 2 mm segment of the renal nerve was gently dissected from the artery and interfaced with a bipolar stainless-steel wire electrode (Bioflex wire AS633; Cooner Wire). A thin layer of medical-grade silicone (Kwik-Sil; World Precision Instruments, Sarasota, FL, United States) was added over the nerve for insulation and mechanical stability. The RSNA was recorded at 1 kHz sampling rate using a Neuro Amp EX (AD-Instruments). The full-wave was rectified and averaged in 1 s intervals for analysis. Baseline values were determined by averaging 30 s of recorded data before DPN stimulation. Baseline RSNA values were considered as 100%, and experimental values were expressed as a change in percentage of the baseline (ΔRSNA,%). Hexamethonium bromide (60 mg/kg), an autonomic ganglia nicotinic acetylcholine receptor antagonist (Touw et al., 1980), was administered intravenously at the end of the experiments to confirm that RSNA signals were recorded from post-ganglionic renal fibers. Neural activity was further confirmed by the abolition of the recorded signal 30-min after euthanasia.

#### Blood Pressure Measurements

For animals in Phase I and II studies, the BP was recorded acutely in anesthetized animals using a wired pressure sensor that was attached to a cannula implanted into the femoral artery. For animals in Phase III, BP was measured using a tail cuff in awake and restrained animals (*n* = 4), or a telemetry BP system implanted into the femoral artery (*n* = 9).

#### Wired Blood Pressure Recording

A 1.5–2 cm incision was made in the inner part of the left leg to expose the femoral artery, where a heparinized (20 IU/ml) cannula (0.6 mm outer diameter) was inserted and secured using 4.0 silk sutures. The cannula was connected to a calibrated pressure transducer (AD-Instruments, MLT1199) and coupled to a bridge amplifier and power supply (AD-Instruments, FE221 and ML826, respectively). BP measurements were obtained continuously during the acute studies and exported at 1,000 and 100 bits per second, respectively. A PowerLab data acquisition system and LabChart Pro software (both from AD Instruments, Colorado Springs, CO, United States) were used to digitalize and visualize the data.

#### Tail-Cuff Blood Pressure Measuring

For non-invasive measurements in fully awake animals, the rats were placed in an animal holder (HLD-RM, Kent Scientific), and a tail-cuff BP system based on volume pressure recording (VPR) sensor technology (CODA, Kent Scientific) was used to measure the systolic and diastolic BP.

#### Telemetry Blood Pressure Monitoring

A wireless BP sensor (HD-S11-F2, DSI Harvard Bioscience, Inc.) was implanted in a cohort of animals in Phase III studies. The device battery was implanted in the abdominal cavity and fixed with sutures to the abdominal walls. The pressure catheter was implanted in the femoral artery. An ambient pressure reference (APR-2, DSI) was used for calibration during measurements (accuracy ± 1 mmHg). The measurements were obtained every 15 s using the PONEMAH software 6.51.

#### Ethics Statement

All protocols and surgical procedures were designed to prevent animal discomfort and suffering. These were approved by the University of Texas at Dallas, University of Texas Southwestern Medical Center, and the University of Houston, Institutional Animal Care and Use Committees, following the guidelines provided by the National Institute of Health (NIH).

#### Spectrogram Analysis

In a small cohort of SHR animals (*n* = 3), MATLAB R2020a was used with a hamming window with a length of 128 samples, 64 samples of overlap, and 128 samples for fast Fourier transform (FFT). A 10-s window from before, during, and after stimulation was selected. Frequencies below 50 Hz were considered related to RSNA and from 50–100 Hz related to stimulation artifact. The intensity of the short-time Fourier transform was averaged over each 2-s window and converted to decibels, divided by the maximum spectrogram frequency to calculate spectral density in dB/Hz.

#### Histology

At the end of the studies, the animals were euthanized with an overdose of sodium pentobarbital (120 mg/kg, intraperitoneal [ip]). The DPN was harvested and fixed in cold 4% paraformaldehyde in phosphate-buffered saline (PBS; pH 7.2) for 24 h, cryoprotected in 30% sucrose, embedded in optimal cutting temperature (OCT) media, and cut in 35 μm cross-sections in a cryostat. The sections were rinsed, blocked, and incubated with primary antibodies as described previously (Gonzalez-Gonzalez et al., 2018). Beta III tubulin (1:400, Sigma, T4026), myelin glycoprotein zero, (P0 1:400; Millipore, AB9352), and the 110 kDa activated macrophages glycoprotein maker ED1 (1:200 Abcam, 31630). Secondary antibodies coupled to Alexa Fluor 488 or 555 (Invitrogen; 1:200 dilution) or Cy5 bis-NHS ester (Jackson ImmunoResearch; 1:400 dilution) were used for visualization. The sections were imaged in a confocal microscope (Nikon, eclipse Ti^®^).

#### Statistical Analysis

Evaluation of pulse duration in voltage-controlled stimulation was achieved by a mixed effect analysis followed by a Sidak multiple comparison test. The effect of stimulation intensity was evaluated using an unpaired Student’s *t*-test. For the spectral density analysis, an ANOVA test was performed with 2-s window from before, during, and after stimulation selected for all frequencies. The intensity (as decibels) was averaged for each time window. To calculate spectral density in dB/Hz, values were divided by the maximum spectrogram frequency (500 Hz). *Post hoc* Tukey’s multiple comparison test was applied to assess pairwise comparison between means. One-way ANOVA followed by Dunnett’s test was used for the evaluation of the BP differences. Statistical analysis was performed using the GraphPad Prism software version 9.1.2 and MATLAB R2020a.

## Results

### Consistent Cardiovascular Depressor Responses Induced by Deep Peroneal Nerve Stimulation

**FIGURE 2:**
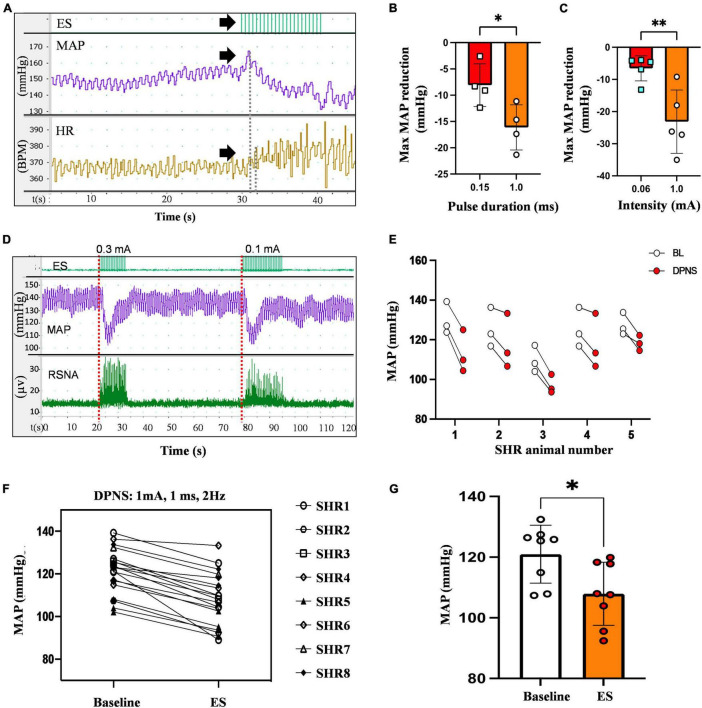
Reduction in MAP by DPNS. **(A)** Stimulation of the DPN at 2 Hz (arrows) evokes a reduction in MAP and increases the SRNA (red dotted lines). **(B)** 1 ms pulse duration and **(C)** 1 mA pulse amplitude induced more effective MAP depressor responses (unpaired t-test; *p < 0.05; **p < 0.008, *n* = 4–5). **(D)** Reproducible MAP reduction evoked by two sequential DPNS trains. **(E)** MAP reduction evoked by three consecutive trains of DPNS in five individual SHR animals. **(F,G)** 1 ms pulses induced a reduction in MAP in each animal and a significant reduction in MAP compared to baseline (unpaired *t*-test; **p < 0.02, *n* = 8). ES, electrical stimulation, MAP, mean arterial pressure; HR, heart rate; BPM, beats per minute; RSNA, renal sympathetic nerve activity; DPN, deep peroneal nerve.

An immediate and reproducible arterial depressor effect was observed with a concomitant increase in heart rate (HR) in response to electrical stimulation of the DPN in anesthetized animals with ventilation support (Figure 2A). Pulse duration, tested with 0.2–0.4 mV stimuli at 2 Hz for 30 s, showed a maximal depressor response with 1 ms pulses (−16 mmHg) as compared to 0.15 ms (−8 mmHg; *p* < 0.05; unpaired *t*-test *n* = 4; Figure 2B). Stimulation intensity tested using monophasic cathodic 1 ms pulses showed a higher depressor response evoked by 1 mA (−23 mmHg) compared that elicited by 0.6 mA stimuli (−7 mmHg, respectively; *p* < 0.005 unpaired *t*-test; *n* = 5; Figure 2C). The DPNS depressor responses were found to be highly reproducible over repetitive stimulations. Figure 2D shows MAP changes induced by two consecutive DPNS stimulation events, and Figure 2E demonstrates consistently evoked depressor responses in 5 individual animals over 3 sequential treatments.

The optimized parameters, 1 mA, 1 ms cathodic monophasic pulses, were used at 2 Hz for 30 s in anesthetized SHR animals without ventilation support to confirm their efficacy. These DPNS parameters efficiently induced a cardiovascular depressor response in all animals (Figure 2F), reducing the average baseline MAP from 108–132 mmHg to 92–120 mmHg (Figure 2G, Student’s *t*-test, *p* = 0.02, *n* = 8).

### Modulation of Renal Sympathetic Nerve Activity by Deep Peroneal Nerve Stimulation

**FIGURE 3:**
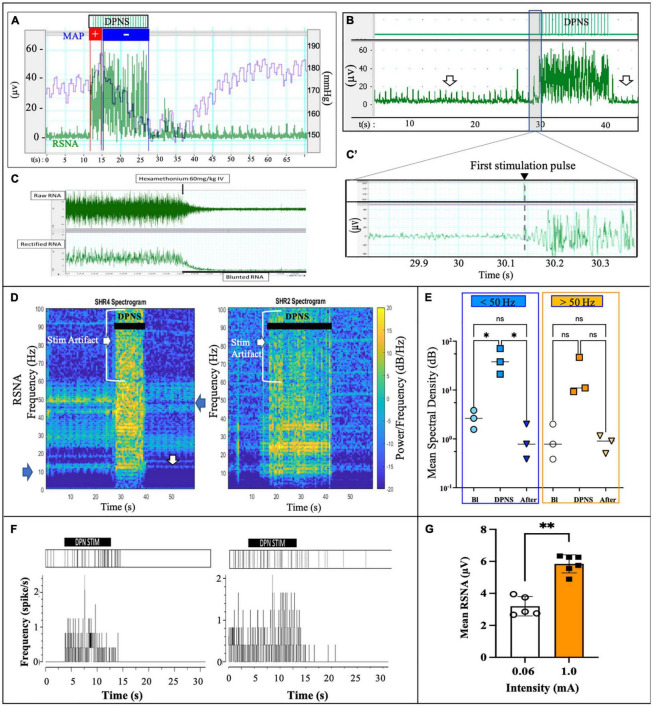
DPNS neuromodulation of RSN activity. **(A)** Spontaneous RSNA (green) increased in amplitude and frequency during the 10 s DPNS. Concurrently, the MAP decreases (blue). **(B,B’)** The onset of RSNA was observed at approximately 0.2 s after the first DPNS stimulation pulse. Arrows point before and after RSNA, to highlight the decrease in amplitude. **(C)** Nicotinic receptor blocker hexamethonium (60 mg/kg) confirmed RSNA activity. **(D)** Power spectral analysis in two different SHR animals after 10 and 20 s of DPNS showed the RSNA band at 10–50 Hz; arrows point signals and stimulus artifact. **(E)** Compared to baseline (Bl), the mean SSD showed a significant increase in the RSNA range (ANOVA *p < 0.05, *n* = 3), but not 10 s after stimulation. **(F)** Raster plots of two waveforms from RSNA, one was evoked during the stimulation and the second was present before stimulation and reduced thereafter. **(G)** 1 mA stimulation pulses increased the amplitude of the evoked RSNA (**p < 0.004, paired *t*-test, *n* = 5). ES, electrical stimulation; DPN, deep peroneal nerve; DPNS, deep peroneal nerve stimulation; RSNA, renal sympathetic nerve activity.

A notable increase in RSNA was observed during DPNS with the similar onset and offset as the electrical stimulation. Overlapping the MAP with the RSNA showed an initial pressor response of about 3 s, followed by a 10 s of continued depressor response, which was reversed to baseline at the end of stimulation (Figure 3A). This activity was different from the stimulation artifact as the onset of RSNA was not synchronous with that of the electrical stimulation, showing a delay of 100–200 ms after the first stimulation pulse (Figures 3B,B’). The ganglion blocker hexamethonium blunted the RSNA activity, demonstrating that these neural signals were sympathetic (Janssen et al., 1989; Figure 3C). Furthermore, the frequency observed in renal nerve activity (10–50 Hz) was differentiated from the 2-Hz stimulus pulses delivered to the DPNS, from the evoked paw movement, and from the stimulation artifact registered above 50 Hz. Power analysis allowed to confirm different signals, unlike the stimulation artifact showed minimal average power density at baseline (0.7 dB/Hz) with some increase during DPNS to 11.2 dB/Hz at frequencies > 50 Hz, the SRNA signals showed regular spiking activity before stimulation and with a power density of 11.2 dB/Hz. This RSNA increased to 37.8 dB/Hz during DPNS (>50 Hz frequency band) and showed a tendency to decrease for 5 s, albeit not significantly compared to baseline (Figures 3D,E). One-way ANOVA followed by Bonferroni’s test showed that these changes in power density were significant only in the low-frequency range (^**^p < 0.001, *n* = 3; Figure 3E). We then used principal component analysis to test if waveforms present at baseline were modulated by DPNS. Two waveforms were identified with evoked activity at 0.5–2.5 Hz and were presented only during the stimulation period. Another one was active before the stimulation and was negatively modulated after the stimulation (Figure 3F). Finally, we looked at peak-to-peak amplitude in the spontaneous RSNA activity before and after DPNS and found that increasing the stimulation current from 0.06 to 1.0 mA significantly increased the amplitude of the evoked RSNA activity 83 ± 8%, from 3.19 ± 0.61 μA to 5.84 ± 0.56 μA (^**^p < 0.004 paired *t*-test, *n* = 5; Figure 3G). Together, these data indicate that DPNS induced changes in RSNA, suggesting a possible role of the renal nerve in the reduction of MAP by this neuromodulation approach.

### Wireless Deep Peroneal Nerve Stimulation Induces a Depressor Response in Awake Animals

**FIGURE 4:**
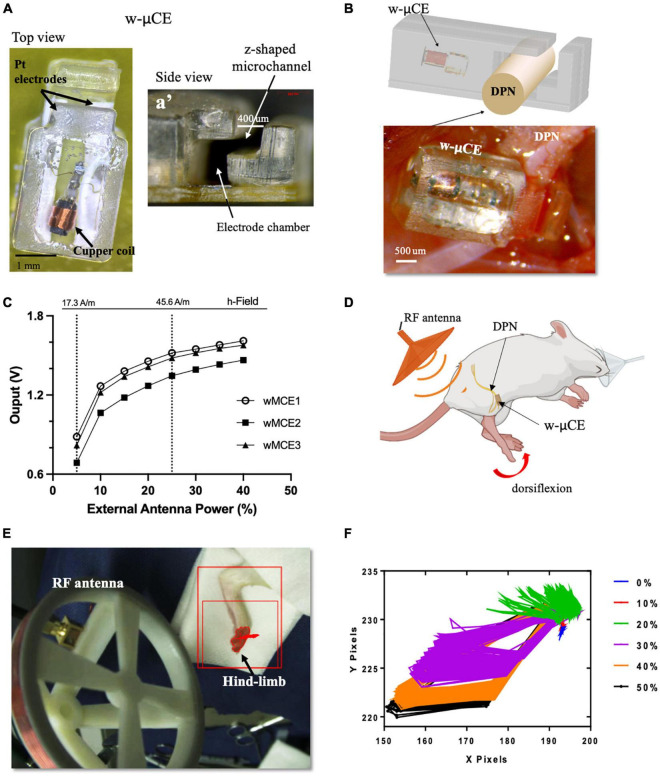
w-μCE for sub-chronic DPNS. **(A)** Picture of the w-μCE top view. **(A’)** Side view of the insertion z-shaped channel and electrode chamber. **(B)** Schematic and picture of the w-μCE implanted onto the DPN. **(C)** Voltage output from the w-μCE as a function of power from the external antenna (*n* = 3). **(D)** Illustration of the device implanted in the DPN and evoked dorsiflexion. **(E)** Placement of the external antenna, 3–4 cm from the w-μCE; the hind-limb was stained to facilitate dynamic pixel tracking during videotaping. **(F)** Pixels tracked from the video recorded during DPNS sessions are displayed in an X/Y graph. The evoked movement was directly proportional to the RF power (0–50%). w-μCE, wireless multichannel electrode; DPNS, deep peroneal nerve stimulation; RF, radio frequency.

Small w-μCE electrodes were implanted onto the DPN of SHR rats and were externally controlled and powered by a radio-magnetic antenna at a carrier frequency of 10.3 MHz (Figure 4A). This device uses a slide-and-lock approach where the nerve passes through a microchannel, 20–30% smaller than the outside diameter of the target nerve, and is placed in an electrode chamber where it expands to its original size, fitting the stimulation chamber tightly (Figure 4a’). The w-μCE was implanted by placing it under the nerve and slightly pulling it upward to stretch it slightly reducing its diameter, allowing it to slide through the Z-shape microchannel and into the electrode chamber (Figure 4B). The output voltage of the w-μCE neural stimulator is a function of the electromagnetic field strength, which is determined by the RF antenna power, and its distance from the implanted device. The devices generated an average of 1.4 V at a 45.6 A/m magnetic field, delivering 0.6–1 mA cathodic pulses at 2 cm from the external antenna (Figure 4C). During active stimulation, the w-μCE on the DPN evoked contraction of the TA muscle producing paw dorsiflexion (Figure 4D). We confirmed that magnetic fields of 17.3–45.6 A/m were able to power the implanted w-μCE devices in 6 SHR animals and videotaped the motor responses produced by DPNS.

The hind-limb was color labeled in the ankles and toes to trace the movement of paw dorsiflexion evoked by the external antenna at 28.16 A/m (Figure 4E) and monophasic cathodic pulses at 2 Hz at 0–50% power intensity. Figure 4F displays the pixels obtained from the video as an x, y trajectory of the limb movements evoked at increasing power levels from the external RF antenna. Threshold limb activity was observed at 20% of power and the maximal response at 40%, with magnetic fields below the 1.6 W/Kg SAR limit.

**FIGURE 5:**
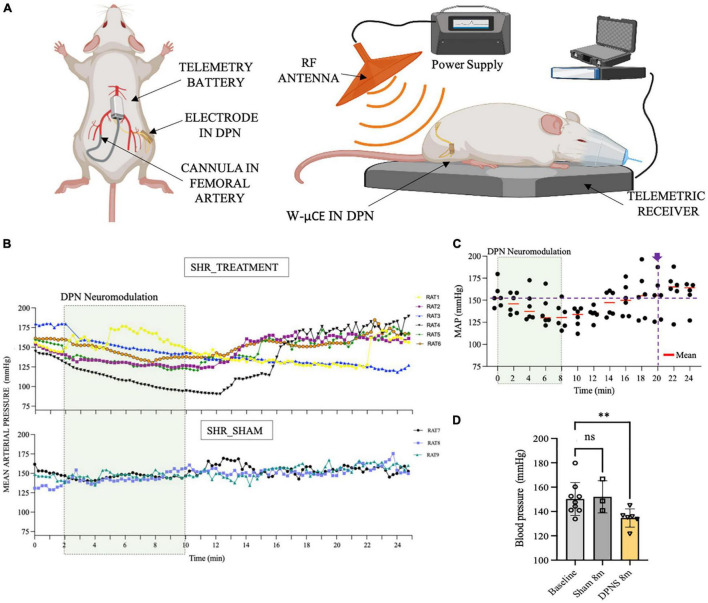
Effect of sub-chronic DPNS on mean arterial pressure in SHR animals. **(A)** Experimental setup; mean arterial pressure was measured with a telemetric device from the femoral artery. The external RF antenna delivered pulses to the w-mCE on the DPN. **(B)** Mean arterial pressure modulation by DPN stimulation, at day 21 of implantation (*n* = 6 SHR animals treated, *n* = 3 SHR sham). At baseline, all animals showed MAP values of (150 ± 14 mmHg; *n* = 9); the shadowed square represents the period of stimulation. SHR treated showed a decrease from 159 to 128 mmHg (*n* = 6), while the sham group maintained a MAP of 150 ± 13 mmHg (*n* = 3). **(C)** Individual datapoints of SHR with an active w-μCE (*n* = 6) with their means obtained every 2 min. The depressor response progressively returned to baseline levels at minute 20 from the start of the stimulation (164 ± 26 mmHg). **(D)** Effect of DPN stimulation at minute 8 significantly reduced the MAP (**p < 0.004 one-way ANOVA followed by Dunnett’s test) in the DPNS-treated group (135 ± 8 mm Hg; *n* = 6), while MAP values were similar in sham animals (150 ± 13 mm Hg; *n* = 3). DPN, deep peroneal nerve; DPNS, DPN stimulation; RF, radio frequency; SHR, spontaneous hypertense rats; w-mCE, wireless micro-channel electrode.

In Phase III, a cohort of 9 SHR animals was also implanted with telemetry BP measurement systems (Figure 5A), evaluated for active DPNS implanted with functional w-μCE stimulators (*n* = 6), and compared to sham-treated animals that are implanted with not-functional devices (*n* = 3). Three weeks after recovery, all animals were exposed to the external RF treatment and telemetry MAP data were recorded before and after the stimulation. The data show averaged MAP per animal at 15-s intervals, demonstrating a decrease in BP in all SHR animals with an active stimulator (DPNS treatment), but not in those with inactive devices and sham treatment (Figure 5B). Active DPNS showed a depressor response that persisted for 12 min before returning to mean baseline values (Figure 5C). At baseline, all animals showed MAP values of 150 ± 14 mmHg (*n* = 9; Figure 5D), but at 8 min after the stimulation, MAP values were similar in sham animals (150 ± 13 mmHg; *n* = 3), but were significantly reduced (*p < 0.04, one-way ANOVA followed by Dunnett’s test) in the DPNS-treated group (135 ± 8 mmHg; *n* = 6; Figure 5D). The depressor response in animals with an active w-μCE was progressively returned to baseline levels 14 min later (164 ± 26 mmHg). Maximal reduction in BP in the DPNS group was observed 2 min after the end of stimulation, which showed a reduction from 159 mmHg prior to stimulation to 128 mmHg after. These data confirmed that stimulation of the DPN using a fully implantable device can reproducibly induce a MAP depressor response.

To evaluate the effect of DPNS treatment in fully awake animals, a separate cohort of four SHR animals was acclimated and stimulated for 10 min while restrained in a cylindrical enclosure, repeated daily for 10 days. In these animals, BP was measured by tail-cuff before and 1 and 2 h after the bioelectronic treatment. Evoked hindlimb movements by DPNS were used to confirm the treatment and showed that it failed in two animals during the first week, but remained effective in the other two animals during the 2 weeks test period.

**FIGURE 6:**
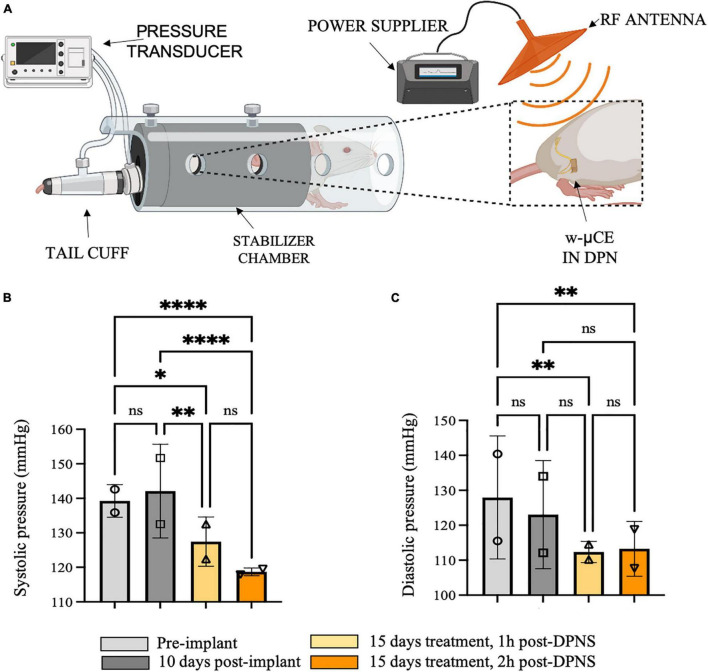
Effect of sub-chronic DPNS on blood pressure in SHR awake animals. **(A)** Experimental setup; blood pressure was measured with a tail cuff in restrained and fully awake animals. The external antenna delivered pulses to the w-mCE on the DPN. **(B,C)** Multiple systolic and diastolic pressure measurements were taken during baseline (i.e., pre-implant and 10 days post-implant) and 15 days after daily 10-min DPNS treatment, at 1 and 2 h post-DPNS (8–19 measurements in two animals, *p < 0.05, **p < 0.001, and ****p < 0.0001). DPN, deep peroneal nerve; DPNS, DPN stimulation; SHR, spontaneous hypertense rats; w-mCE, wireless micro-channel electrode.

This group of animals showed BP measurements comparable with previous groups with telemetric device implantation, where the systolic blood pressure average was 139 ± 5 mmHg and 10 days after recovery was 142 ± 1 mmHg (Figure 6A). Fifteen days after daily DPNS significantly lowered the systolic (Figure 6B) and diastolic (Figure 6C) BP, at 1 and 2 h post-electrical stimulation (128 ± 7 and 119 ± 1 mmHg; respectively) when compared to baseline. The reduction in systolic BP was estimated at −10% at 1 h and −16% at 2 h after the DPNS when compared to the post-implantation baseline. These changes were found to be significant using a two-ANOVA followed by Tukey’s multiple comparison test despite the low sample number, given that 15–20 measurements of MAP were taken per animal at each time point.

### Gross Anatomy and Histology

**FIGURE 7:**
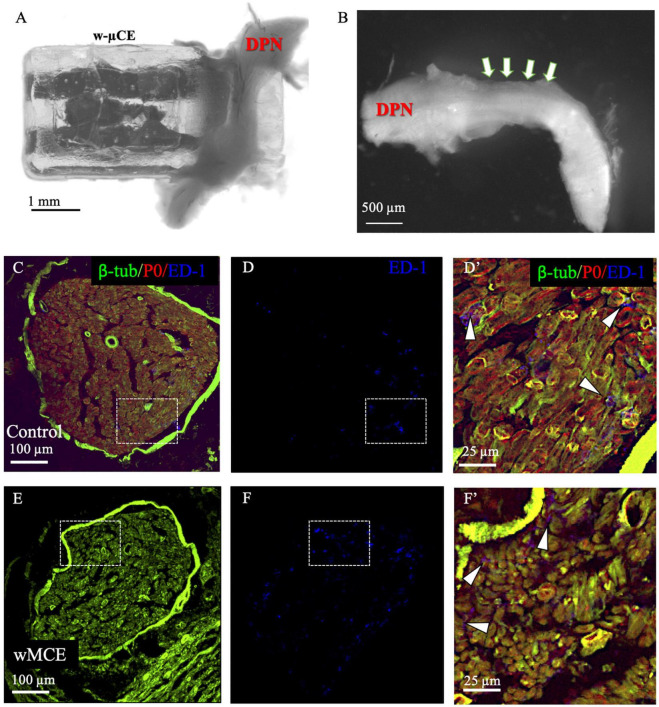
Nerve histology after sub-chronic wMCE implantation. **(a)** Picture of DPN in the device 30 days after implantation. **(b)** Nerve segment after device removal, arrows point out the area where the electrode was implanted. Cross-section histology of control **(c–d’)**, and wMCE implanted DPN **(e–f’)**, **d’** and **f’** are magnifications of the dotted line squares in panel **c,d** and **e,f**, respectively. Fluorescent labels: b-tubulin (axons), P0 (myelin), ED-1 (activated macrophages).

The foreign body response to the w-μCE on the DPN was evaluated in four SHR animals 30 days after implantation. The gross histological observation did not show signs of hematoma, inflammation, nerve compression, and tissue damage in the area of the implanted device. The nerve appeared normal and the device was not displaced despite being placed near the knee, an area of high mobility (Figure 7A). After device removal, the nerve had a normal and healthy appearance (Figure 7B). This agrees with observations of these animals during the 2-week testing, that showed normal gait behavior, and no signs of pain or discomfort during normal walking or after stimulation were noted, despite the clear observation of evoked paw movement.

The DPN tissue was processed for immunofluorescence to visualize axonal marked b-tubulin, and the myelin protein P0 marker, in the control non-implanted side (Figures 7C,D’) when compared to the side implanted with the w-μCE device (Figures 7E,F’). Cross sections of the nerve segment implanted in the middle of the device showed either normal shapes or slight deformations when compared to the normal contralateral side. Axon and myelin staining showed an absence of nerve fiber loss or demyelination in the implanted nerve, being comparable to the non-implanted side. We also stained the tissue with antibodies that label activated macrophage (ED-1) markers to evaluate the possibility of inflammation. The numbers of activated macrophages per cross-section range were estimated at 44 ± 20 ED1 + cells and were similar to that seen in not-implanted controls (*n* = 4 each group, *t*-test *p* < 0.01). Together, the histological evaluation of the tissue indicated that placement of this miniature wireless stimulator did not cause harm to the nerve.

## Discussion

Cardiovascular depressor responses have been previously reported after stimulation of the sciatic nerve or its deep peroneal branch (Baum et al., 1966; Kim and Romero-Ortega, 2012). This report further elucidates that pulses of 1 ms duration are more effective when compared to shorter ones in mediating the reduction of MAP and that the depressor response can be reproduced with sequential stimulation events in anesthetized and ventilation-assisted animals. We also confirmed in anesthetized animals without breathing support, that DPNS was lowered 12 mmHg (*p* < 0.02) in MAP, suggesting that this effect is independent of respiratory-sympathetic pathways. While this is a tempting interpretation, the interaction between the cardiovascular and respiratory systems during DPNS is certainly complex and deserves further investigation. Long stimulation pulses are known to recruit more axons, particularly of medium and large diameters, including those that mediate antidiuresis in the renal nerve, when compared to short pulses or increased amplitude, which recruit small unmyelinated axons involved in renal vasoconstriction (DiBona et al., 1996). This observation suggests that increasing both amplitude and pulse duration, additional renal axons are recruited. The renal nerve is known to play an important role in cardiovascular homeostasis (Ricksten et al., 1979; Veiga et al., 2021), and it contains both efferent sympathetic and afferent sensory axons from the kidney to the dorsal root ganglia, which project centrally to the brain (Leal et al., 2012; Mizuno et al., 2014; Pettigrew et al., 2017). It has been reported that denervation of the sinoaortic baroreceptors results in an immediate increase in RSNA, HR, and MAP (Dibona and Jones, 2001). In this study, DPNS induced an initial increase in RSNA during the first 2–3 s, followed by a reduction in renal activity and MAP, despite the increase in HR. The observed activation of the RSNA during the DPNS was not expected since its activity is associated with hypertension (DiBona and Kopp, 1997; Günter et al., 2019; Osborn et al., 2021) and is known to be overactive in SHR animals (Lundin et al., 1984). We further determined that the evoked RSNA activity was not a stimulation artifact, as it was asynchronous and delayed when compared to the first electrical pulse of the DPNS. Given that DPNS also induced paw movements that might be contaminating the RSNA recordings with motion or EMG artifacts, we did a power spectral analysis to separate the 2 Hz stimulation frequency from higher frequency signals. At high frequencies (i.e., 50–100 Hz) we only observed the stimulation artifact during DPNS, with comparable power density before or after the stimulation. In contrast, the spontaneous RSNA activity in the 10–50 Hz band was increased significantly to maximal power density during DPNS. This likely reflects the induced changes in RNSA, as its spiking frequency contains several frequency bands, including 0.2–0.4, 2–6 Hz (cardiac and baroreceptors) and 10–12 Hz (Malpas, 1998). The high-frequency RSNA does not correlate with other functions, appears after baroreceptor denervation, and seems to have a central origin (Malpas and Ninomiya, 1992). Thus, these signals seem related to the functional regulation of the tubules, blood vessels, and the juxtaglomerular granular cells in the kidney, which are normally supplied by different post-ganglionic neurons with bimodal axon diameter distribution (1.2 and 1.6 μm) (Dibona, 2001).

The modulation of MAP during the DPNS is likely to be a complex interplay between the direct stimulation of small myelinated Aδ fibers and unmyelinated c-fibers likely mediating the immediate increase in RNSA (Murphy et al., 2011), and central command signals that regulate the sympathetic outflow are evoked by mechanical and metabolic changes in the muscle (Malpas, 1998), such as the activation of baroreceptors known for phasic inhibitory input to the sympathetic ganglia (Kumada et al., 1990). This interpretation is supported by reports of electrical stimulation of the central afferent axons in the renal nerve, shown to induce an immediate reduction in sympathetic nerve activity and MAP (Aars and Akre, 1970). Therefore, a plausible mechanistic explanation of the relation between RSNA modulation and the reduction in MAP by DPNS might involve the initial pressor response activating the baroreceptors, which in turn reduced the sympathetic tone and mediated the subsequent reduction in MAP (Moffitt et al., 2005). However, the precise role of RSNA in MAP during DPNS, and the role of baroreceptor reflexes and RSNA activity, in response to DPNS reduction in MAP warrants further investigation.

This study also reported that DPNS can be elicited using fully implantable w-μCE electrodes anchored to the nerve through a Z-shaped microchannel. Stretching the nerve during device implantation did not cause apparent nerve injury, likely due to the unique elastic properties of peripheral nerves. Transient 5–10% strain to the nerve can cause minor alterations in nerve conduction that can be recovered immediately with no functional deficits (Rickett et al., 2011), and they can stretch up to 40% briefly without causing damage (Rydevik et al., 1990; Wall et al., 1992; Yeoh et al., 2021). In humans, extensive studies of stress and strain on peripheral nerves have indicated that 20–32% of elongation does not result in structural and functional damage (Sunderland and Bradley, 1961). This explains the fact that the w-μCE electrode implanted in the DPN for up to 30 days did not cause discomfort to the animals, and no signs of nerve injury or inflammation were observed in histological preparations.

The wireless w-μCE stimulators allowed the simultaneous evaluation of DPN neuromodulation therapy and telemetric acute BP measurements in animals 3 weeks after implantation. These animals were anesthetized so that the distance to the antenna and the stimulation could be controlled. Wireless stimulation of the DPN induced a reduction in MAP only in hypertensive animals implanted with active stimulators, but not in sham animals with inactive devices, confirming that DPNS induced an acute depressor response in animals with sub-chronic implants and that the application of RF field by the external antenna alone, did not affect the BP. We also confirmed that the DPNS induced a depressor response in restrained fully awake animals, despite the small variabilities in placing the external antenna. In these animals, systolic BP and diastolic BP were reduced for up to 2 h after DPNS, demonstrating the feasibility of this neuromodulation modality. Importantly, none of these animals showed signs of pain or discomfort during the stimulation and evoked paw movements. This is in agreement with the low content of nociceptor fibers in this nerve and the low stimulation intensity used in this study.

## Limitations

This study is limited to the evaluation of the acute and sub-chronic effects of DPNS. Further research is necessary to investigate whether BP regulation can be achieved chronically and whether continuous activation of the DPN is needed to maintain the beneficial effects of this bioelectronic treatment. It is important to note that SHR animals are not representative of all forms of human hypertension but rather are a model of essential hypertension only. Whether neural stimulation of the DPN can be effective in other types of hypertension remains to be determined.

In summary, this study reports the feasibility and effectiveness of peripheral nerve stimulation for successfully reducing the BP in SHR animals, offering support to the notion that this neuromodulation modality may be beneficial in alleviating RH.

## Data Availability Statement

The original contributions presented in the study are included in the article/supplementary material, further inquiries can be directed to the corresponding author.

## Ethics Statement

The animal study was reviewed and approved by the IACUC at The University of Texas at Dallas.

## Author Contributions

MR-O, SS, and WV designed the research and acquired funding for the project. MR-O and CB contribute to device fabrication. MR-O, MG-G, KR, DVL, DL, JB, H-KK, and AK: *in vivo* studies. AH-R did device characterization. MR-O and MG-G did data analysis. MR-O, MG-G, SS, and WV contributed with data interpretation. MR-O and MG-G wrote the manuscript. All authors discussed the results and revised the manuscript.

## Conflict of Interest

MR-O owns shares in RBI Medical, a medical device company. RBI Medical did not have any role in data collection, analysis, or the manuscript. The remaining authors declare that the research was conducted in the absence of any commercial or financial relationships that could be construed as a potential conflict of interest.

## Publisher’s Note

All claims expressed in this article are solely those of the authors and do not necessarily represent those of their affiliated organizations, or those of the publisher, the editors and the reviewers. Any product that may be evaluated in this article, or claim that may be made by its manufacturer, is not guaranteed or endorsed by the publisher.
